# Effect of Microindentation on Electroluminescence of SiC P-I-N Junctions

**DOI:** 10.3390/ma15020534

**Published:** 2022-01-11

**Authors:** Tingwei Zhang, Adrian H. Kitai

**Affiliations:** 1 Department of Materials Science and Engineering, McMaster University, Hamilton, ON L8S 4L7, Canada; 2 Department of Engineering Physics, McMaster University, Hamilton, ON L8S 4L7, Canada

**Keywords:** SiC, microindentation, electroluminescence

## Abstract

The influence of microindentation on the electroluminescence of silicon carbide was studied in forward-biased 4H SiC p-i-n junctions. Four spectral regions at approximately 390, 420, 445 and 500 nm initially observed on virgin samples strongly depend, in regard to magnitude, on the condition of the starting die. These spectral regions may be interpreted as arising from either phonon-assisted band-to-band transitions or from defect-related transitions. The same SiC die were then subjected to mechanical damage brought about by a series of closely spaced microindentations directed approximately perpendicular to the c-axis. The spectra taken after a first set and subsequently a second set of microindentations are distinct from the initial spectra in all cases, and differences are interpreted as being due to the modification of existing defects or additional defects being generated mechanically. The influence of microindentation on the ideality factor is measured and discussed. Measured light flux with respect to a standard light source is also shown at each microindentation stage.

## 1. Introduction and Background

Due to the nature of its strong covalent bonding, relatively high thermal conductivity and wide bandgap, SiC is commonly used for high-temperature, high-radiation and high-power applications. It is also known for a low formation energy difference between polytypes, resulting in more than 200 SiC polytypes with distinct stacking sequences [1]. Among all the SiC polytypes, the most commercially available are 3C-SiC, 4H-SiC and 6H-SiC [2]. The properties for each polytype can be seen in Table 1 [3]. In spite of the identical chemical composition of each SiC polytype, they have distinct electronic properties.

The recent achievement of high quality 8-inch vapor-grown SiC wafers was motivated by the electric vehicle and sustainable power generation sectors [4]. SiC is also used as the substrate material for high-performance GaN light-emitting diodes (LEDs) [5]. It is a historical fact that SiC is one of the oldest LED materials, and it played a role in the discovery of the LED [6]. The indirect bandgap of SiC in which radiative recombination is forbidden prevents its use as an active LED material. However, visible radiation can still be observed in virtually all forward-biased SiC junctions, owing to deviations from the perfect lattice caused by phonons, point defects, line defects, stacking faults and surfaces. 

The study of SiC p-n or p-i-n junction electroluminescence provides valuable insight into the recombination mechanisms in SiC and can help identify defects [7]. In 4H-SiC material, a peak at 390 nm that is commonly observed from the spectral data is associated with phonon-assisted band-to-band recombination. The indirect bandgap of a non-faulted 4H-SiC has a corresponding band-edge wavelength of 380 nm [8], where the 10 nm longer emission wavelength can be explained by well-studied SiC phonon energies on the order of 100 meV [9].

From the literature, three additional peaks located at 420, 460 and 480 nm have been detected that provide evidence of recombination at various stacking faults [10,11,12]. Similarly, in other previous work, peaks at 390, 424 and 540 nm were observed from the PL mapping of a 4H-SiC sample. A structural analysis by scanning TEM confirmed the existence of stacking faults [13]. The peak at 424 nm indicates single Shockley stacking fault formation, and the 540 nm peak is attributed to the band edge recombination from 3C-SiC polytype inclusions inside the 4H-SiC matrix, considering that the bandgap of the 3C polytype is 2.36 eV. 

In addition to experimental results, modeling by using Density Functional Theory also can help us analyze the change of radiative recombination energy resulting from stacking faults in 4H-SiC [14]. Stacking faults may behave similarly to quantum well structures [15], where electrons get trapped and recombine with a corresponding emission of sub-bandgap photons [16]. Due to the ease of formation, stacking faults are known to cause a serious problem for device stability, as they can also be induced at high current density during operation [17]. The EL data in an experiment showed a sharp 424 nm peak, along with a broad green emission peaked around 530 to 540 nm. In this case, recombination-induced stacking faults (RISFs) were found to be responsible for the 424 nm peak, whereas the broad green emission range was caused by neighboring partial dislocations [18]. 

The identification of such fluorescent defect centers allows further study of defect-induced device degradation or future applications, such as quantum computing and single photon emitters [19,20]. In this work, commercially available 4H-SiC p-i-n junctions from a SiC transistor device were studied, and microindentation was performed on one side of the p-i-n junctions. The effects of both grown-in defects and subsequent defects introduced by mechanical damage were studied. The recombination mechanism corresponding to the EL spectrum peaks is discussed, along with a series of electrical characterizations of device performance. 

## 2. Sample Preparation and Measurements

Three 4H-SiC junction transistors purchased from GeneSiC (GA20JT12-263) (Dulles, VA, USA) were mounted into epoxy pucks (Electron Microscopy Sciences, Hatfield, PA, USA) with connections to the base-collector p-i-n junction for convenience of holding and clamping during the surface exposure for optical and electronic testing. A smooth and optically clear die edge surface close to the active junction area was obtained after grinding into the original package by using SiC sandpaper (Metlab, Niagara Falls, NY, USA) with grits between 800 and 1200 for the final polish. Samples 1, 2 and 3 were oriented differently during grinding and polishing. Inevitably, samples were subjected to unavoidable heat and mechanical stress due to the soldering, grinding and the polishing process. Light emitted from the exposed die surface of each sample was measured and analyzed.

**Figure 1:**
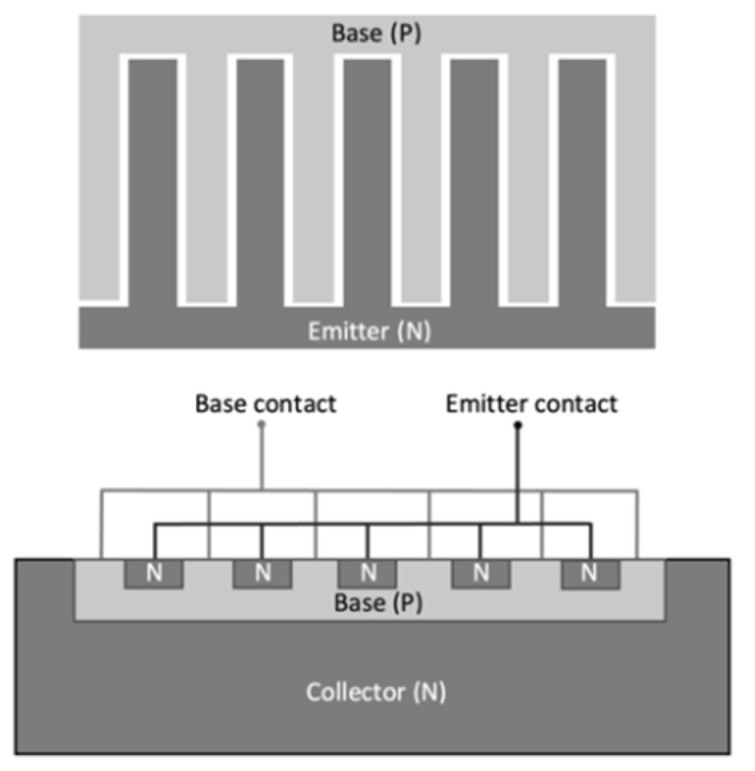
A typical interdigitated structure of a bipolar junction transistor.

Each die consists of an NPN bipolar junction transistor with interdigitated base and emitter electrodes, as shown in Figure 1. Inside our sample, the *p*-type base and *n*-type emitter intersect with each other periodically with electrode connection on the top of each junction and a *n*-type collector underneath the interdigitated structure. Samples 1 and 2 were exposed at the direction perpendicular to those interdigitations, whereas Sample 3 was exposed from the direction parallel to such a structure. Specifics of the geometry of the exposed die surface are later described in detail for each sample.

EL spectra were obtained by using a grating spectrometer Optometrics Corp. DMC1-03 (Littleton, CO, USA) with an avalanching photodiode Hamamatsu S12053 (Hamamatsu, Japan) operated at 158V reverse bias. Data were taken at room-temperature ambient conditions, with 0.2 A current flow across the base-collector junction. Samples were then subjected to indentation by using a micro-hardness tester (CLEMEX Vickers micro-hardness tester, Longueuil, Canada) to generate a first stage of microindentations to the polished sample edge and near the surface of the sample where the interdigitation is located. After repeating the measurement of spectra, each sample was subjected to a second set of microindentations (see Table 2 for details). 

**Figure 2:**
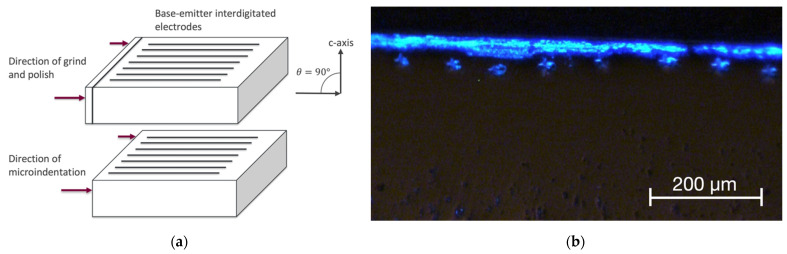
(**a**) A simplified diagram of the die structure to show grinding direction, viewing angle and indentation direction of Sample 1. (**b**) Optical microscopy image of Sample 1 after second-stage microindentation.

Sample 1 was ground and polished in a direction on an edge face perpendicular to the interdigitations and the c-axis of the 4H SiC die, as shown in Figure 2. The sample was not ground deep into the active junction area. Visible-light EL emission can be seen from the sample in the form of a light stripe, which corresponds to the edge view of the active region at the junction. The first and second stages of microindentations are also shown, appearing as a series of green emission points near the emission stripe. 

**Figure 3:**
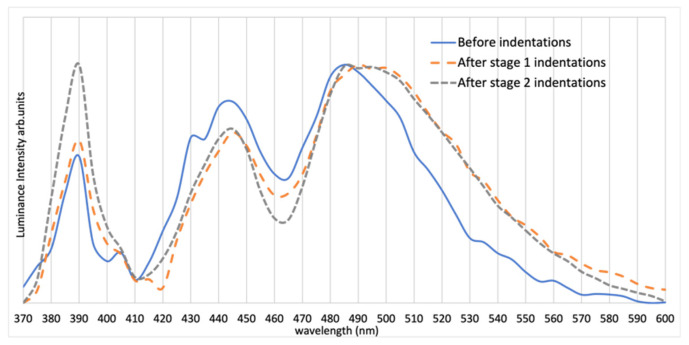
Spectra of Sample 1 before indentations and after Stages 1 and 2 of indentations. Note the change in the spectrum particularly after Stage 1 of indentations (spectra are normalized at the maximum value). All sample were connected to a DC power supply with 0.2 A current flow.

The spectrum of Sample 1, as shown in Figure 3, indicates two strong peaks located at 390 and 445 nm and a long wavelength peak that is broadened and ranges from 480 nm to 500 nm, depending on the indentation condition. 

**Figure 4:**
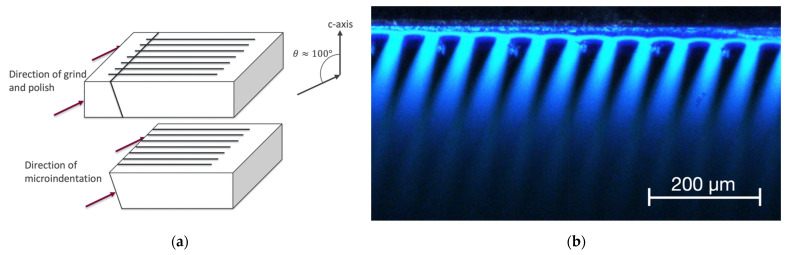
(**a**) A simplified diagram of the die structure to show grinding direction, viewing angle and microindentation direction of Sample 2. (**b**) Optical microscopy image of Sample 2 after second-stage microindentation.

**Figure 5:**
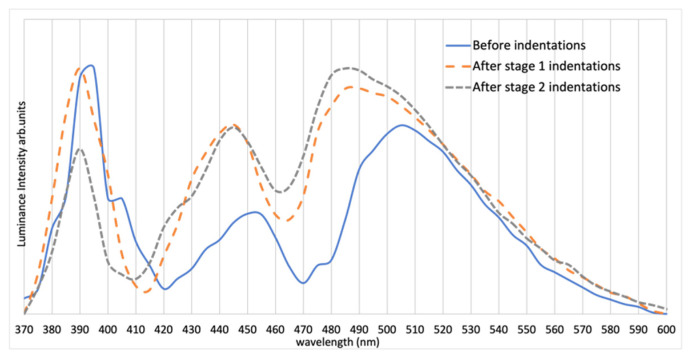
Spectrum of Sample 2 before and after indentation at each stage (normalized at highest peak).

**Figure 6:**
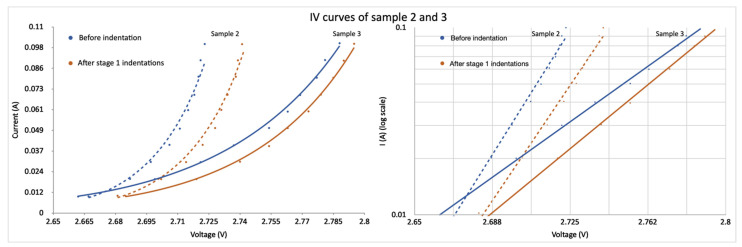
IV curves of Samples 2 and 3. Dashed lines show IV data from Sample 2 before and after first indentation stage, and solid line corresponds to Sample 3. Notice the change of slope between samples in the log plot.

The polished face of Sample 2 was ground and polished in a direction tilted at 100 degrees relative to the c-axis, as shown in Figure 4, and the interdigitated structure can now be seen in the optical micrograph as light stripes, due to this slightly tilted angle relative to an edge face. The micro-hardness tester load was reduced to 200 gf for Sample 2. As in Sample 1, the spectrum of Sample 2, shown in Figure 5, also indicates two strong peaks located near 390 and 445 nm and a third broadened green peak located around 480 to 500 nm. In addition to spectral measurements, the diode ideality factor and relative intensity data were collected to further analyze the influence of microindentation. For Sample 2, the ideality factor n increased from 0.95 before indentations to 1.02 after Stage 1 and to 1.13 after Stage 2. This was calculated based on exponential regression of the IV graph, as presented in Figure 6. Notice that, at a high current, the temperature starts to affect IV characteristics of Sample 2, shown as a steeper line, whereas, in Sample 3, the IV characteristic continues to follow an exponential regression. The relative light flux emitted from the partly exposed SiC die was obtained by using an integrating sphere (Newport, Irvine, CA, USA). To enable a valid comparison of relative light flux between samples, a reference LED light source (Cree, Durham, NC, USA) was measured in the integrating sphere, and these reference data were used to correct for experimental inconsistencies when switching samples in the integrating sphere.

**Figure 7:**
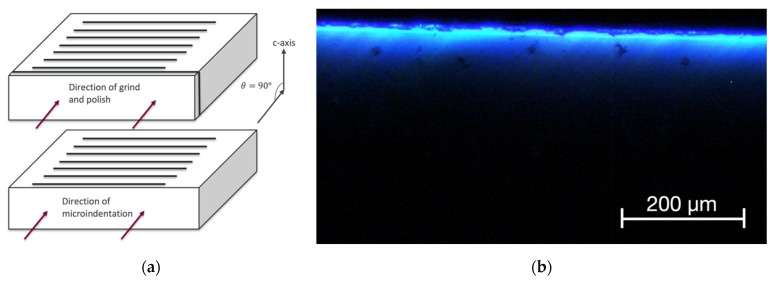
(**a**) A simplified diagram of the die structure to show grinding direction, viewing angle and indentation direction of Sample 3. (**b**) Optical microscopy image of Sample 3 after second-stage microindentation.

**Figure 8:**
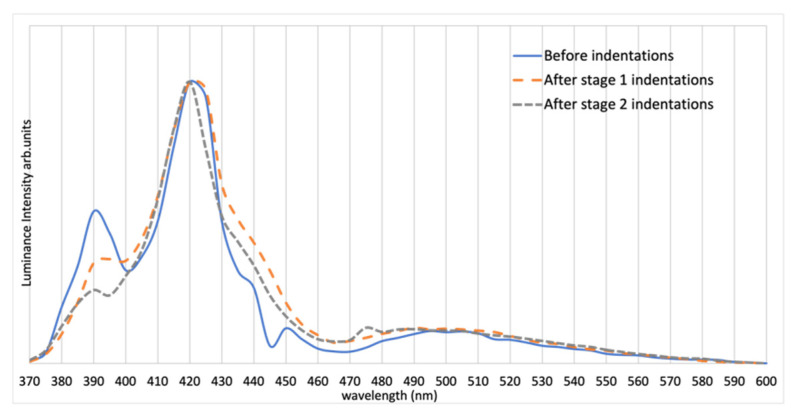
Spectrum of Sample 3 before and after indentation at each stage (normalized at highest peak).

Sample 3 was edge-polished in a direction with $\theta =90^{{}^{\circ}}$ relative to the c-axis and parallel to the interdigitations, as shown in Figure 7. Figure 8 shows a strong EL peak of Sample 3 at 420 nm, suggesting the formation of single Shockley stacking faults [17]. Sample 3 underwent 200 gf tester load during microindentation. The spectra and ideality factor were obtained for this sample. Selective I–V data of Sample 3 are presented in Figure 6. The ideality factor *n* of Sample 3 before indentation is 2.12. However, the voltage reading sometimes fluctuated over a 100 mV range during the I–V testing after both Stages 1 and 2, especially after second-stage indentation, thus making the interpretation of the result somewhat unreliable. The IV data of Sample 3 after first-stage indentation in Figure 6 were taken when the device was stable. The instability of SiC junctions with stacking faults is well-known [21]. The light-flux data of Sample 3 are presented in Table 3. 

## 3. Discussion

**Figure 9:**
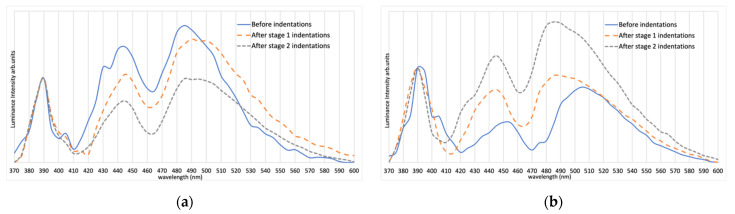
Spectrum of Samples 1 (**a**) and 2 (**b**) before and after indentation at each stage (normalized at 390 nm).

**Figure 10:**
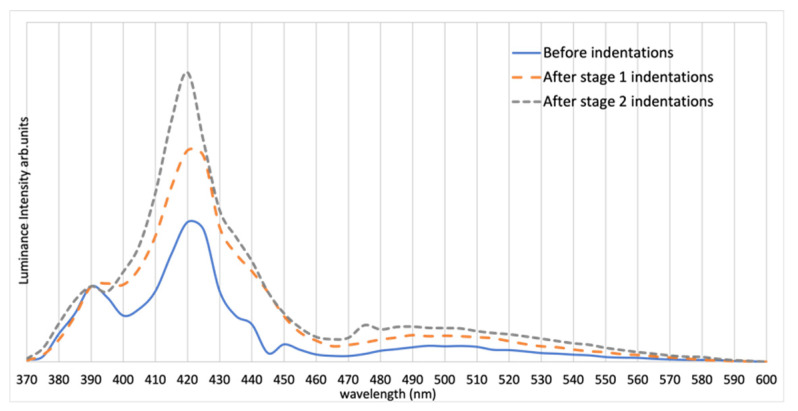
Spectrum of Sample 3 before and after indentation at each stage (normalized at 390 nm).

The spectra of Samples 1 and 2 show peaks at 390 and 445 nm, along with a third 480–500 nm broad green emission peak. While the 390 nm emission indicates a phonon-assisted band-to-band recombination [9], the exact mechanisms of the other peaks remain somewhat speculative. From Reference [17], we note that peaks at 455, 480 and 500 nm are evidence of quadruple Shockley SFs (4SSFs), triple Shockley SFs (3SSFs) and double Shockley SFs (2SSFs), respectively. Although there is no direct evidence to indicate that the observed peaks are originated from stacking faults, it would still be a valid assumption that there may be a stacking-sequence variation in the vicinity of the interdigitated junction areas. To further analyze the change of spectra due to microindentation, we normalized our spectra relative to the 390 nm band-to-band recombination for each sample, as shown in Figure 9 and Figure 10. 

In general, either plastic deformation along the slip direction or a brittle fracture across the cleavage plane results in microscopic defects being generated in SiC when a sufficient external stress is applied [22]. When the temperature is below the brittle-to-ductile transition temperature (T_BDT_), a limited plastic deformation driven by the partial dislocation motion is expected [23]. Dislocation nucleation and extended stacking faults were observed previously by others under TEM testing at the condition below the brittle-to-ductile temperature [24]. Meanwhile, a photoluminescence test result of 6H-SiC from Weifang Lu et al. has also shown that carbon-related surface defects would also increase the emission in the green region [25] which assembles microcracks if SiC underwent brittle fracture. Based on the normalized figure above, those changes of structure due to either microcracks, dislocation motion or a combination of both have caused the broadening of the green-emission region in both Samples 1 and 2. 

In Figure 9, the relative intensity of the peak at 445 nm and the broadened green region of Sample 2 have increased noticeably relative to the band-to-band recombination after microindentations. The increase of intensity shown in Table 3 supports the increase of emission in the green region in Sample 2, since there is no other source of increased light flux. For Sample 1, however, the change of relative intensity of 445 nm and green-emission region to the band-to-band recombination shows the opposite result. This may be due to Sample 2 having a tilted angle (see Figure 4), but it is more likely owed to the fact that Sample 2’s surface was ground deeper into the die edge, entering the active region of the junction. The mechanically induced defects would therefore have more influence within the junction area and would increase the localized defect-driven green emissions. Moreover, the difference between the peak location before and after indentation may also indicate a shift of stacking sequence which could be induced by the external shear stress [17].

In addition to these observations, each as-received SiC sample may possess distinct defect types and structure at the junction interface, and this could be an additional explanation as to the distinctions in their spectra, considering they were not manufactured for LED application. Indications of pre-existing stacking fault formations have been observed as varying from sample to sample in our previous work [26].

Evidence of this hypothesis is from Sample 3. Despite having unstable IV data, the spectrum of Sample 3 shows a dominant 420 nm peak at all microindentation stages. This 420 nm peak is independent of the polish direction. As mentioned before, it is known that stacking faults constitute a prominent defect in SiC and can be active as a quantum well structure with an ideality factor greater than 2 [27]. An interesting observation from the normalized spectrum is that, after more indentations are formed, the 420 nm peak becomes more significant with respect to the 390 nm peak, with an increased overall intensity (shown in Table 3) after the first indentation stage. One way to interpret this is that the band-to-band recombination became less favored when more defects were introduced into the system, or the stress applied during microindentations extended the pre-existing stacking faults [24,28]. In addition, the broadening of the green emission after each stage is also relatively small compared to the other two samples. 

**Figure 11:**
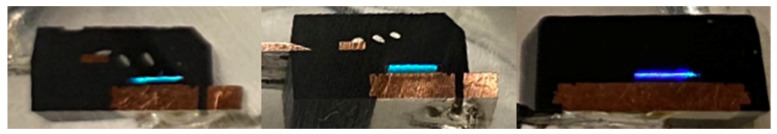
Image of Samples 1, 2 and 3 electrically connected in series and operating at 200 milliamps, showing differences of geometry and color. All samples are shown after second-stage indentations.

A camera image of all three samples is shown in Figure 11, revealing how the differences of the spectra affect the color from sample to sample. 

## 4. Conclusions

Mechanically induced defects by using microindentation were introduced to three 4H-SiC samples. EL spectra were studied at each stage of the microindentation. All three samples had a broadened green emission after each indentation stage, while having distinct intrinsic spectra and intensity data. Sample 3, with a strong 420 nm peak, indicates that stacking-fault-associated recombination dominated the overall radiative recombination. Most of the observed changes to the samples can be explained by the microscopic defects generated during the microindentation process that favor or suppress specific regions of light emission based on the known literature modeling of SiC defects. The diode-ideality-factor results are consistent with a substantial increase in defect-mediated recombination after microindentation.

## Figures and Tables

**Table 1:** Electronic properties of SiC polytypes [3].

Property	3C-SiC	4H-SiC	6H-SiC
Bandgap E_g_ (eV), 300 K	2.36	3.26	3.02
Electron mobility $\left({\mathrm{cm}}^{2}V^{-1}s^{-1}\right)$	1000	//*c*-axis:1200; ⊥*c*-axis: 1020	//*c*-axis: 100; ⊥*c*-axis: 450

**Table 2:** Details of sample preparation.

Sample Number	Direction	θ	Load (Gram-Force)	Number of Stage 1 Indentation	Total Number after Stage 2 Indentation
Sample 1	Die edge perpendicular to interdigitations	90	300	7	13
Sample 2	Edge perpendicular to interdigitations	100	200	11	22
Sample 3	Edge parallel to interdigitations	90	200	11	22

**Table 3:** PV reading and intensity ratio of Samples 2 and 3.

Sample Number		Before Microindentation	After Stage 1	After Stage 2
Sample 2	Relative light flux	1.00 *	1.77 approx	2.28
Sample 3	Relative light flux	1.00 *	2.70	2.29

* Relative brightness is normalized to the reading before microindentation for each sample.

## Data Availability

The data presented in this paper are available upon request.
